# Metformin, mitochondria and cancer

**DOI:** 10.1186/2049-3002-2-S1-O27

**Published:** 2014-05-28

**Authors:** Navdeep S Chandel

**Affiliations:** 1 Department of Medicine, Feinberg School of Medicine, Northwestern University, Chicago, IL, 60611, USA

## 

Most cancers have intact mitochondria function and we previously showed that mitochondria metabolism and ROS is essential for tumorigenesis. However, there are a subset of cancers which arise from mutations which abolish activity of TCA cycle enzymes, including Fumarate Hydratase (FH) deficient renal cancer cells. While oxygen consumption is severely reduced in FH deficient human cancer cells, we uncovered that they are still dependent on mitochondrial metabolism and ROS for cell proliferation. Based on these results we propose that mitochondrial metabolism and/or ROS could be an attractive target for therapy. We will discuss the role of mitochondria in regulating cancer and how metformin might exert its anti-tumorigenic properties by inhibiting certain aspects of mitochondrial function.

